# Clinicopathological characteristics of De Garengeot hernia: six case reports and literature review

**DOI:** 10.1186/s40792-020-01098-z

**Published:** 2021-01-11

**Authors:** Shigeaki Tsuruta, Hideo Miyake, Hidemasa Nagai, Yuichiro Yoshioka, Norihiro Yuasa, Masahiko Fujino

**Affiliations:** 1 Department of Gastrointestinal Surgery, Japanese Red Cross Nagoya First Hospital, 3-35 Michishita-cho, Nakamura-ku, Nagoya, 453-8511 Japan; 2 Department of Cytology and Molecular Pathology, Japanese Red Cross Nagoya First Hospital, 3-35 Michishita-cho, Nakamura-ku, Nagoya, 453-8511 Japan

**Keywords:** De Garengeot hernia, Femoral hernia, Appendix

## Abstract

**Background:**

De Garengeot hernia, wherein the appendix is present within a femoral hernia, is a rare disease; therefore, the clinicopathological features remain to be clarified. This study aimed to reveal the clinicopathological characteristics of De Garengeot hernia.

**Case presentation:**

Six patients who underwent appendectomy and herniorrhaphy between 1999 and 2018 were included. The incidence of De Garengeot hernia was 3.2% among the 182 femoral hernias that required surgery during the study period. The median age of the patients was 78 years, and five patients were women. The median body mass index was 20.1. Patients frequently had fever or elevated CRP level. Preoperative diagnoses based on computed tomography were femoral (*n* = 3), inguinal (*n* = 2), and De Garengeot (*n* = 1) hernias. Emergency and elective surgeries were performed in four and two patients, respectively. Histopathological examination of the resected appendix showed gangrenous appendicitis (*n* = 3), perforated appendicitis (*n* = 2), and appendiceal ischemia (*n* = 1) in the patients. Postoperatively, one patient developed sepsis.

**Conclusions:**

Preoperative diagnosis of De Garengeot hernia is often difficult, and patients frequently have severe appendicitis. Precise diagnosis is required, and emergency surgery should be considered depending on the severity of appendicitis.

## Background

De Garengeot hernia is diagnosed when the content of a femoral hernia is the appendix [1]. Preoperative diagnosis remains challenging, as it is a rare disease. In addition, surgical procedures depend on the preoperative diagnosis, severity of appendicitis, and abscess formation. There are limited reports of more than three cases of the condition collectively in a single institution [2–4]; therefore, the clinical characteristics and problems associated with diagnosis and treatment have not been fully clarified.

## Case presentation

A review of the prospective hernia database in our department identified six cases of De Garengeot hernia (3.2%) among 182 cases of femoral hernia that required herniorrhaphy during a 20-year period between January 1999 and December 2018. Table 1 presents the background and clinicopathological factors including age, sex, body mass index (BMI), body temperature, white blood cell (WBC) count, serum C-reactive protein (CRP) level, preoperative diagnosis, surgical procedures, histopathological findings of the resected appendix, postoperative complications, and hospital stay.

**Table 1:** Patient background and clinicopathological factors

Case	1	2	3	4	5	6
Age	92	67	71	88	85	71
Sex	Female	Female	Female	Female	Female	Male
Body mass index	16.6	23.5	23.9	18.3	21.9	15.8
Body temperature (°C)	nv	nv	37.0	37.9	39.1	38.0
White blood cell count (/μL)	7000	nv	7900	8700	15,300	5800
C-reactive protein (mg/dL)	nv	nv	7.44	11.98	20.09	4.4
Signs of bowel obstruction	−	−	−	+	−	−
Preoperative diagnosis	Femoral hernia	Femoral hernia	Femoral hernia	Inguinal hernia	Inguinal hernia	De Garengeot hernia
Timing for operation	Emergent	Within 24 h	Emergent	Emergent	Emergent	within 24 h
Approach	Inguinal	Inguinal	Inguinal	Inguinal and abdominal	Inguinal and abdominal	Inguinal
Abscess in the hernia sac	−	−	−	+	−	+
Operative methods	Mesh plug	McVay	McVay	McVay	McVay	McVay
Pathological findings of the appendix	Gangrenous appendicitis	Ischemia	Gangrenous appendicitis	Perforated appendicitis	Gangrenous appendicitis	Perforated appendicitis
Postoperative complications	−	−	−	Peritonitis, sepsis, DIC	SSI	-
Postoperative hospital stay (days)	9	10	8	29	23	8

*DIC* disseminated intravascular coagulation, *SSI* surgical site infection, *nv* not varidated

Continuous variables were expressed as mean ± SD or median (IQR) as appropriate. Normality was assessed by Shapiro–Wilk test. We used JMP version 10 (SAS Institute Inc., Cary, NC, USA) to perform statistical procedures.

### Patient background

The median age of the patients was 78 years (interquartile range: IQR 70–89) and five patients were women. The median BMI was 20.1 kg/m^2^ (IQR 16.4–23.6). The median body temperature was 38.0 ℃ (IQR 37.2–38.8) in four patients whose records were available. The median WBC count was 7900/μL (IQR 6400–12,000). The median CRP level was 9.7 mg/dL (IQR 5.2–18.1) in four patients whose records were available.

### Preoperative diagnosis base on CT

**Fig. 1:**
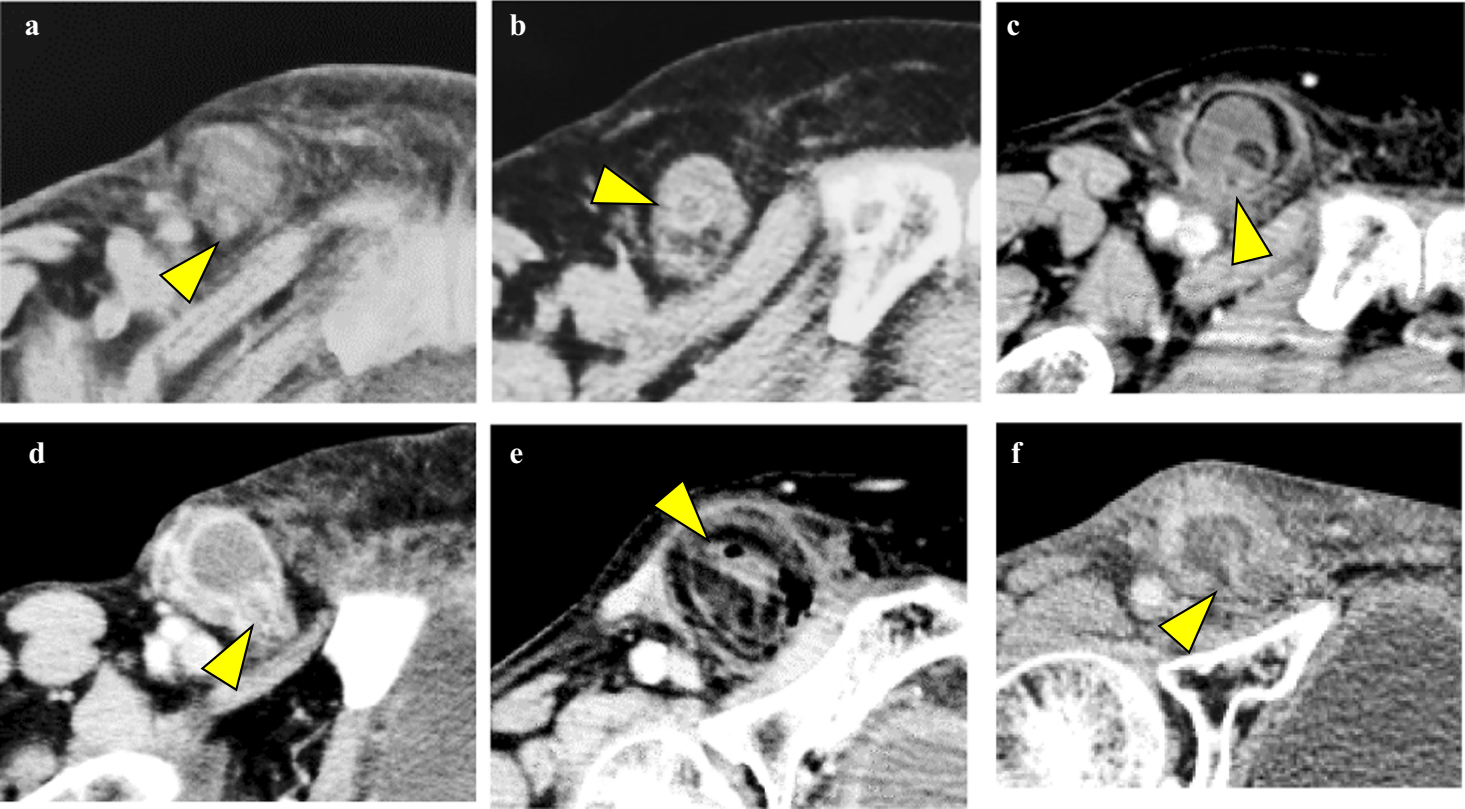
Axial images of contrast enhancement CT showing a tubular structure (arrowhead) surrounded by or along with high/iso/low-density masses on the ventral and medial sides of the femoral vein in Cases 1–6 (**a**–**f** in this order). **d**, **f**: A low-density ovoid lesion with high-density capsule suggestive of an abscess around the appendix. **e**: A honeycomb-like low-density mass surrounding a tubular structure suggestive of the greater omentum

**Fig. 2:**
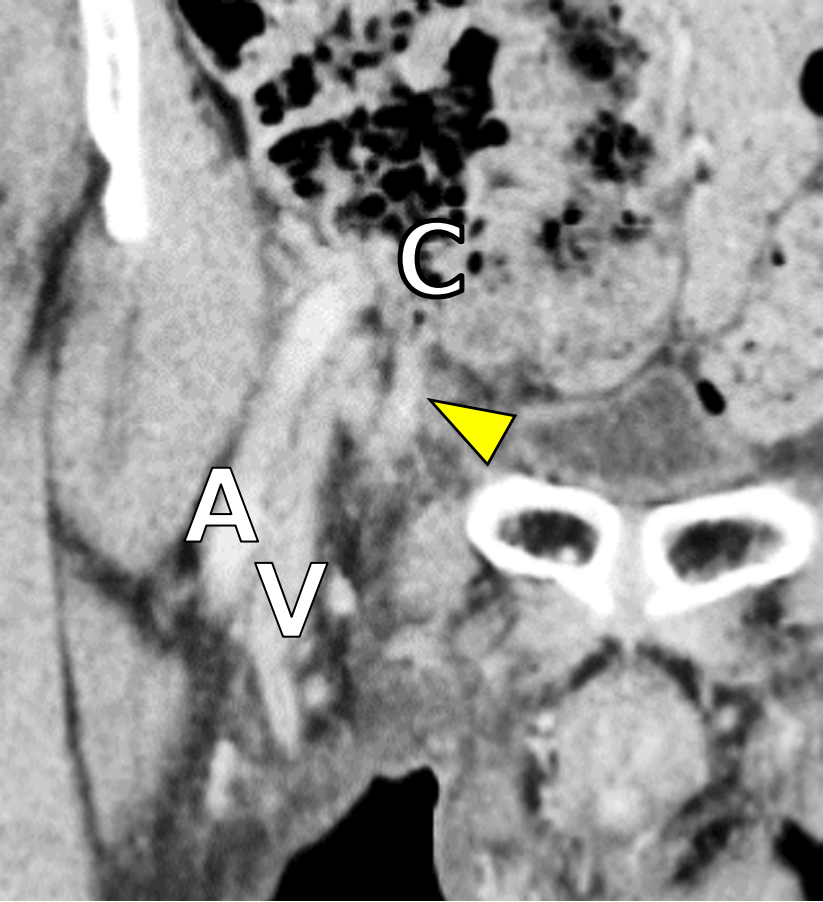
Coronal image of contrast enhancement CT showing a blind-ended tubular structure (arrowhead) on the medial side of the femoral vein, which is continuous with the cecum (C). A: femoral artery, V: femoral vein

Preoperative computed tomography (CT) was performed in all patients (Fig. 1), which revealed femoral (*n* = 3), inguinal (*n* = 2), and De Garengeot (*n* = 1) hernias. Retrospective review of the CT images indicated a tubular structure surrounded by or along with high/iso/low-density masses on the ventral and medial sides of the femoral vein in all patients (Fig. 1a–f). Figures 1f and 2 show CT images of Case 6, which could be preoperatively diagnosed as De Garengeot hernia. The axial image shows a low-density ovoid lesion with a high-density capsule and a tubular structure on the ventral and medial sides of the femoral vein, which indicates an abscess and the appendix, respectively (Fig. 1f). On the other hand, the coronal image shows an isodense blind-ended tubular structure originating from the cecum (Fig. 2).

### Surgical procedure

Emergency surgery was performed in four patients and elective surgery (within 24 h of presentation) was performed in two patients. The surgical approach was inguinal in all cases; however, an additional abdominal incision was made in two patients. Intraoperatively, an abscess was observed within the hernia sac in two patients (cases 4 and 6, Fig. 1d, f). Appendectomy was performed in all patients. Herniorrhaphy was performed by the McVay method in five patients and the mesh plug method in one patient.

### Histology of the resected appendix

**Fig. 3:**
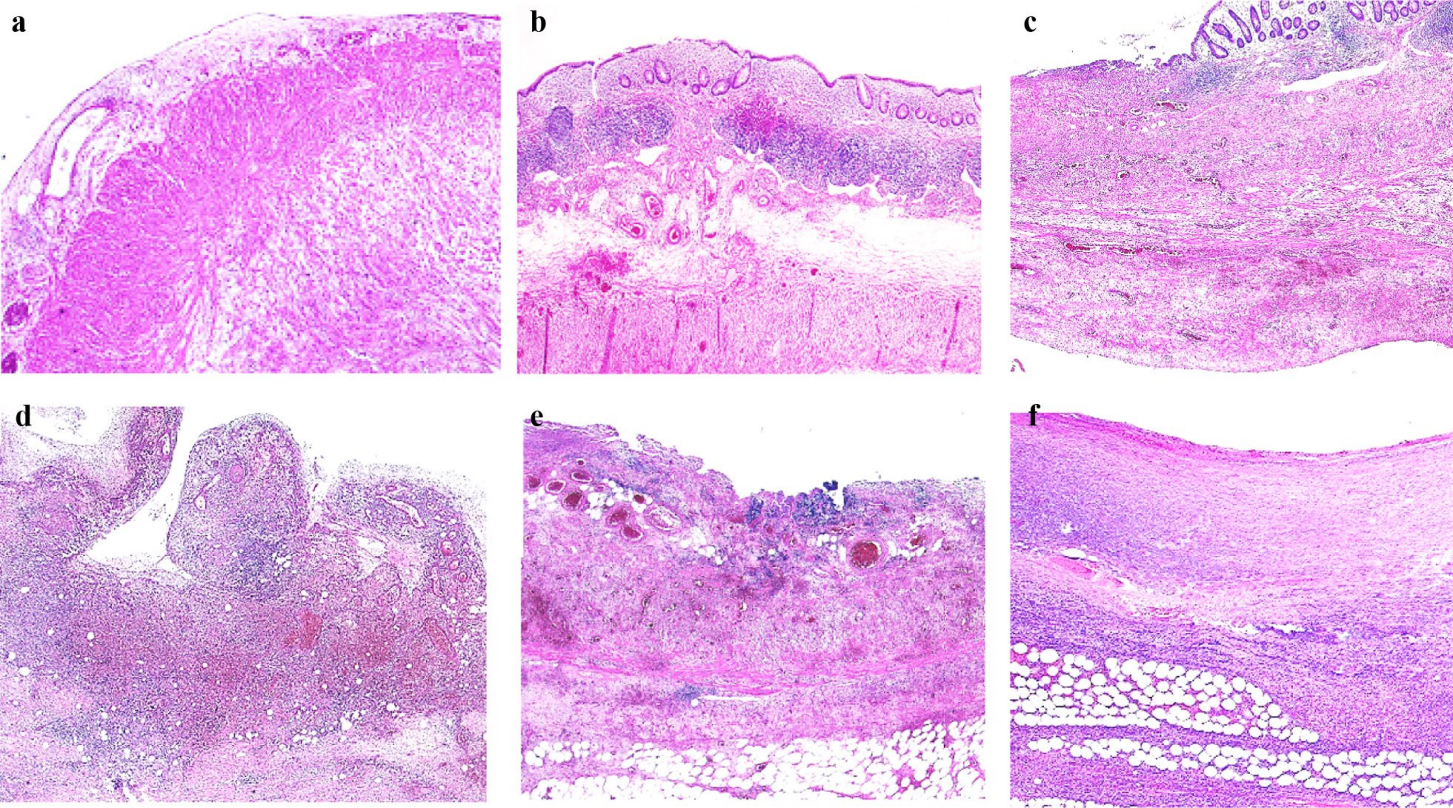
Histopathological images of the appendix in cases 1–6 (**a–f** in this order). Inflammatory cells invading the appendiceal wall with destruction of the wall architecture indicating gangrenous appendicitis (**a, c, d–f**). Macroscopically, the appendix was perforated in cases 4 and 6 (**d**, **f**). Venous dilatation and thrombosis indicate appendiceal congestion (**c**, **e**). Venous thrombosis and submucosal edema indicate ischemia (**b**)

Histopathological examination of the resected specimens showed gangrenous appendicitis, wherein inflammatory cells invaded the appendix with destruction of the wall architecture in three patients, perforated appendicitis in two patients, and appendiceal ischemia in one patient (Fig. 3).

### Postoperative course

One patient with perforated appendicitis and diffuse peritonitis developed postoperative sepsis and disseminated intravascular coagulation (case 4). Surgical site infection (SSI) was observed in one patient (case 5), and the median length of postoperative hospital stay was 10 days (IQR 8–25), without in-hospital death.

## Discussion

This study showed the clinicopathological findings of six cases of De Garengeot hernia, and revealed that the condition occurred frequently in elderly women. Furthermore, the hernia was often associated with fever and elevated CRP levels. Precise diagnosis of the condition was rare and elective surgery was performed occasionally. One-third of the patients had perforated appendicitis. Postoperative complications included SSI and sepsis, which prolonged the duration of hospital stay.

The incidence of De Garengeot hernia has been reported as 0.15–5% of all femoral hernias [3, 5–7]. Forward movement of the appendix into the femoral canal, possibly due to the mobile cecum, the giant cecum extending into the pelvis, or increased abdominal pressure has been hypothesized as the cause of De Garengeot hernia. Obstruction of the appendiceal lumen and impaired blood circulation or venous congestion is likely to induce bacterial growth within the lumen and cause ischemic changes in the appendiceal wall. The pathological findings of the six cases in this study supported this mechanism.

Our study showed that the preoperative diagnostic rate of De Garengeot hernia was insufficient (17%, 1/6), which is in line with that reported in previous studies [8, 9]. This could be due to the rarity of the disease, affecting the ability of the surgeon to interpret the CT images. However, increased diagnostic ability has been observed due to a recent surge in the recognition of cases of De Garengeot hernia by surgeons as well as advances in CT technologies, including thin-slice, coronal, and/or sagittal images. There are three key points in the CT diagnosis of De Garengeot hernia. First, a tubular structure should be identified on the ventral and medial sides of the femoral vein. Second, the tubular structure should be continuous with the bowel (cecum) in the abdominal cavity. Third, the tubular structure should have a blind end.

Our extensive search of Japanese literature (1996–2020) revealed 45 cases of De Garengeot hernia, including the patients in our case series (Table 2) [10–47]. The median age of the patients was 77 years (IQR 71–83), and 84% were women. The mean BMI was 18.2 ± 2.8. The most frequent symptom was a mass in the groin followed by groin pain, and signs of bowel obstruction were infrequent. The median WBC count was 6238/μL (IQR 8195–12,128), and median value of CRP was 2.2 mg/dL (IQR 0.6–6.5). Increased WBC and CRP levels could be markers to estimate the severity of appendiceal inflammation and/or abscess formation. Correct preoperative diagnosis was made in 47% of the patients by CT and/or ultrasonography (US) [13, 17, 20, 24, 26, 29, 30, 32, 33, 37–46]. Emergency surgery was performed in 88% of the patients. The McVay procedure was most commonly performed (43%) [10, 13, 14, 16, 17, 19, 21, 23, 29, 30, 34, 35, 37, 44], followed by the mesh and plug [12, 18, 22, 25, 27, 33], Kugel patch [28, 40, 45, 46], suture of the femoral canal techniques [11, 15, 24, 47], and laparoscopic procedures including the transabdominal preperitoneal [39, 43] and totally extraperitoneal approaches [26, 32]. The advantages of the laparoscopic approach lie in the ability to explore the content of the hernia and reduce the incarcerated organs under direct vision. Furthermore, the procedure is minimally invasive [48]. Intraoperatively, periappendiceal abscess and appendiceal perforation were found in 22% [13, 15, 17, 20, 21, 34, 41] and 9% [14, 17] of the patients, respectively. The incidence of perforation was similar to that reported in a study by Linder et al. on 90 patients reported English literature [9]. Histopathological diagnoses of the resected appendix were gangrenous appendicitis, congestion/ischemia, catarrhal appendicitis, and phlegmonous appendicitis in 44%, 20%, 18%, and 16% of the patients, respectively. Thirteen percent of the patients developed postoperative SSI [11, 14, 15, 17, 19], which led to prolongation of hospital stay.

**Table 2:** Forty-five cases with De Garengeot hernia reported in Japanese literature

Age	Median 77 (IQR 71–83)
Male:Female	7:38
Body mass index	Median 20.4 (IQR 18.1–21.7)
Laterality of hernia (right:left)	45:0
Symptom	
Groin mass	35 (78%)
Groin pain	22 (49%)
Abdominal pain	5 (11%)
Bowel obstruction	2 (4%)
White blood cell count (/μL)	9052 ± 4075
C-reactive protein (mg/dL)	2.2 (IQR 0.4–6.5)
Correct preoperative diagnosis	21 (47%)
Diagnostic modalities	
CT	38 (84%)
US	23 (51%)
X-p	2 (4%)
MRI	1 (2%)
Surgical emergency	
Emergent	37 (88%)
Elective	5 (12%)
Surgical procedure	
McVay	19 (43%)
Mesh and plug	7 (16%)
Kugel patch	4 (9%)
Suture of the femoral canal	4 (9%)
Prolene hernia system/ultrapro hernia system	3 (7%)
Transabdominal preperitoneal approach (TAPP)	2 (5%)
Totally extraperitoneal approach	2 (5%)
Staged surgery (mesh/TAPP)	2 (5%)
Moschcowitz repair	1 (2%)
Intraoperative findings	
Periappendiceal abscess	10 (22%)
Appendiceal perforation	4 (9%)
Appendiceal pathology	
Congestion\Ischemia	9 (20%)
Catarrhal appendicitis	8 (18%)
Phlegmonous appendicitis	7 (16%)
Gangrenous appendicitis	20 (44%)
Chronic appendicitis	1 (2%)
Postoperative complication	
Surgical site infection	6 (13%)
Sepsis	1 (2%)
Postoperative hospital stay (days)	Median 9 (IQR 7–12)

There is no standard approach for the treatment of De Garengeot hernia. Appendectomy and concurrent herniorrhaphy are the treatments tailored based on severity of the appendicitis, patient condition, and surgeon’s preference. Mesh material has not been recommended in the presence of abscess and/or perforation, and staged surgery, laparoscopic appendectomy, or hernioplasty via the anterior approach could be an option in such cases [48, 49].

Hence, Amyand hernia is defined as an inguinal hernia, containing the appendix within the hernia sac. Because De Garengeot hernia and Amyand hernia have each clinical characteristic, the clinical differences among De Garengeot hernia, femoral hernia and Amyand hernia are presented in Table 3 [50–52]. The incidence is comparable between De Garengeot hernia and Amyand hernia; however, the rate of appendicitis is higher in De Garengeot hernia than Amyand hernia (80% in 45 cases of our review in Japanese literature and 92.8% in 222 cases of worldwide literature review [52]). Of note, Guenther TM et al. reported that the incidence of any one of following gross appearance of the appendix including necrosis, perforation, abscess, or fistula was 42% [50]. De Garengeot hernia is female elderly predominant, while Amyand hernia is often associated with male elderly. Preoperative differential diagnosis can be made by image modality including CT: When a blind-ending tubular structure continuous with the cecum is located on the ventral and medial sides of the femoral vein within the femoral canal, the diagnosis is De Garengeot hernia. On the other hand, the tubular structure is located within the inguinal canal, the diagnosis is Amyand hernia.

**Table 3:** Clinical difference among De Garengeot hernia, femoral hernia and Amyand hernia

	De Garengeot hernia	Femoral hernia	Amyand hernia
Hernia orifice	Femoral canal	Femoral canal	Internal inguinal ring or posterior of the inguinal canal
Hernia content	Appendix	Any organ	Appendix
Incidence	0.15–5% in femoral hernia	< 10% of all groin hernias	1% of inguinal hernia
Incidence of appendicitis	80–92.8%	–	0.1%
Age	Elderly	Elderly	Neonates and elderly
Sex	Female predominant	Female predominant	Male predominant
Blood examination	elevated WBC and CRP in case with appendicitis	not specific	WBC and CRP inconsistently associated with the status of the vermiform appendix
CT findings	A tubular structure: (1) On the ventral and medial sides of the femoral vein, (2) Continuous with the bowel (cecum) in the abdominal cavity, (3) With a blind end	Abdominal organ on the ventral and medial sides of the femoral vein	A tubular structure on medial or lateral sides of the inferior epigastric vessels within the inguinal canal
Surgical emergency	Often required	Often required	Inidicated if the appendicitis is preoperatively diagnosed

Guenther TM et al. proposed a classification of De Garengeot hernia based on gross appearance of the appendix [50]. It will prompt an adequate selection of surgical procedures. However, preoperative evaluation of the severity of appendiceal inflammation is more important. If preoperative image modality indicate inflammation of the appendix, especially necrosis and perforation of the appendix, immediate surgery without using mesh such as open repair (McVay) is recommended. When image modality suggest absence of inflammation of the appendix and the patient is clinically stable, planned surgery of laparoscopic repair using mesh such as transabdominal preperitoneal approach is recommended. Cavigli et al. reported a case of De Garengeot hernia in which US showed thickened wall and hypervascularization of the appendix and hyper echoic omental fat, suggesting inflammation of the appendix without necrosis [53]. The thickness and layers of the appendiceal wall can be evaluated with US. In addition, Doppler US can reveal vascularity of the appendix.

The present case reports has several limitations. First, despite recording the data of six patients with this rare disease, several clinical factors including body temperature, WBC count, and CRP levels could not be obtained due to the prolonged study duration. Second, multi-detector CT was introduced in 2007 at our institution, which enabled presentation of finer images as well as coronal and sagittal images. However, three patients who were included in the early study period did not undergo this investigation. As described previously (Fig. 2), the diagnostic sensitivity can be improved by increasing the effectiveness of CT image acquisition and reconstruction [54]. Third, we retrospectively reviewed preoperative CT images; however, US images were not fully investigated due to limited experience. The advantages of US are that the technique is non-invasive and repeatable, and shows superior discriminability due to high spatial resolution. US could facilitate correct preoperative diagnosis of De Garengeot hernia and assessment of the severity of appendicitis [53, 55, 56].

## Conclusion

De Garengeot hernia must be suspected and CT images must be carefully interpreted in patients with a groin mass and pain, as well as elevated WBC count and CRP level. De Garengeot hernia is often associated with severe acute appendicitis, and emergency surgery should be considered depending on the severity of appendicitis.

## Data Availability

Data sharing is not applicable to this article.

## Abbreviations

CT: Computed tomography
BMI: Body mass index
WBC: White blood cell
CRP: C-reactive protein
SSI: Surgical site infection
IQR: Interquartile range
US: Ultrasonography
